# Outcome measures for assessing change in children with hypermobility-associated conditions and chronic lower limb musculoskeletal pain: a Delphi survey of international health professions

**DOI:** 10.1007/s10067-025-07504-x

**Published:** 2025-05-28

**Authors:** Rachal Quinlan, Luke M. Davies, Kelly Gray, Verity Pacey

**Affiliations:** 1 Department of Health Sciences, Faculty of Medicine, Health and Human Sciences Macquarie University, Macquarie University, 75 Talavera Rd, Sydney, NSW G8152109 Australia; 2 School of Primary and Allied Health, Monash University, Frankston, Australia

**Keywords:** Chronic leg pain, Children, Delphi, Hypermobility, Outcome measure

## Abstract

**Introduction:**

The study aims to reach an expert consensus and rank the top outcome measures that health professionals could utilise to measure change in pain, function, fatigue, and quality of life (QoL) of children with hypermobility-associated conditions and chronic musculoskeletal lower limb pain.

**Methods:**

A three-round modified Delphi survey was conducted between February and April 2024. In the first two rounds, experienced health professionals were invited to rate the importance of outcome measures assessing change over time in children (5 years and older) with hypermobility and chronic musculoskeletal lower limb pain. Outcome measures were collated from the literature and participant suggestions and assigned to four domains: pain, function, fatigue, and QoL. A priori threshold of consensus was 70% or greater of participants rating an outcome measure as important or essential. In the third round, participants ranked the outcome measures that met consensus in order of importance.

**Results:**

Overall, 44 health professionals, from 13 countries, participated. Retention rate from round one to round three was 85%. Nine outcome measures achieved consensus.

**Conclusions:**

Reaching consensus on this core set of outcome measures to assess change over time represents a step toward the development of international guidelines for the management of children with hypermobility and lower limb pain.

## Introduction

Generalised joint hypermobility in children and adolescents is the presence of excessive range of motion in multiple joints. Approximately one in five children with hypermobility experience symptoms such as joint instability, poor coordination and proprioception, as well as musculoskeletal pain [1–3]. The Beighton score is a commonly used, easy to administer, valid screening tool to identify generalised joint hypermobility in children [4]. The working threshold for identifying generalised joint hypermobility in children was recently concluded as a Beighton score of 6 or more out of 9, with a score of 7 or more out of 9 considered likely more appropriate during childhood [5]. A recently developed paediatric-specific diagnostic framework for joint hypermobility encourages fluid categorisation of children, from 5 years of age until skeletal maturity, into groups describing phenotypic and symptomatic presentation [6]. This framework supports differentiating between young people with hypermobility as an isolated physical trait, those with musculoskeletal issues related to hypermobility, and those who may develop hypermobility-associated conditions [6].

Children with hypermobility-associated conditions may experience multi-systemic complaints, functional impairments, and varied levels of disability [7]. Lower limb pain, with soft tissue injuries and pain post exercise, is frequently reported in children with hypermobility-associated conditions [8, 9]. Furthermore, a longitudinal study of musculoskeletal pain in school children found that children with hypermobility-associated disorders were more likely to develop chronic pain than non-hypermobile peers [10]. Symptomatic joint hypermobility is reported in many studies to impact a child’s quality of life, participation, and psychosocial well-being [11–13].

In clinical practice, various methods exist to assess the range of symptoms children with hypermobility-associated conditions may experience. A recent systematic review of outcomes used in the literature to assess change in children with hypermobility-associated conditions and lower limb symptoms reported a lack of consensus [14]. No agreed-upon or recommended set of outcome measures currently exists, and 20 different instruments were identified as primary outcome measures across the constructs of pain, function, fatigue, and quality of life [14]. The heterogeneity of outcome measures currently used in studies including children with hypermobility-associated conditions has limited the ability to synthesise data for meta-analysis across several recent systematic reviews of treatment effectiveness [3, 15, 16]. This highlights the importance of achieving consensus on a set of outcome measures, considering the four constructs of pain, function, fatigue, and quality of life, to improve consistency in assessment [14].

Therefore, the aim of this study was to reach consensus and rank the outcome measures that could be utilised in clinical practice to measure pain, function, fatigue, and quality of life in children with hypermobility-associated conditions and chronic musculoskeletal lower limb pain.

## Materials and methods

### Design

An international modified three-round e-Delphi was conducted between February and April 2024. The Delphi technique was used to reach consensus on this particular subject as research is limited and knowledge is indefinite in this clinical area [17–19]. Previously established recommendations and methodological criteria for reporting Delphi studies, in accordance with the Guidance on Conducting and Reporting DELphi Studies (CREDES), were used to guide the study (supplementary 1) [20]. Based on prior research, the a priori threshold of consensus was set at 70% of participants rating an outcome measure as important or essential [21–24]. An electronic survey developed by the research team was placed on Qualtrics (www.qualtrics.com) to collect data over three rounds. The research team pilot tested the survey to ensure comprehension and flow. Ethics approval was obtained from the Human Research Ethics Committee at Monash University (#39,862).

### Participants

International experts in the field of paediatric hypermobility were recruited via patient advocacy groups such as the Ehlers-Danlos Society and professional networks of the research team. Through this purposeful sampling process, a combination of 85 medical, nursing, and allied health professionals with current contact details was selected. Participants were required to be registered health professionals actively treating or having undertaken research on paediatric patients (5 years and over) with hypermobility and chronic lower limb pain within the last 3 years. All participants were required to understand English and provide consent to participate.

Prior to completing the first round, participants were sent a personal email, including a detailed description of the project aims and the Delphi process, and an invitation link to the online survey. In the first round, basic demographic information, including age, gender, country, profession, years of experience, workplace healthcare setting, and consent to participate in the surveys, was collected. All three survey rounds were separated by a 2-week interval. To encourage non-responders to complete the survey, three email reminders were sent (1 week before the response deadline, 4 days before the response deadline, and on the day of the deadline). Those who did not complete the survey or submitted an incomplete survey were not invited to complete future rounds.

### Outcome measures

Twenty outcome measures were sourced from a scoping review, conducted in 2020, investigating outcome measures used in prospective and longitudinal studies to assess change over time in children with generalised joint hypermobility and associated lower limb symptoms (supplementary 2) [14]. To ensure currency, a literature search was conducted on 20/12/23 in three databases (Cinahl, Embase and PubMed), with the Covidence platform used to streamline the review of 1399 additional articles; although no further outcome measures were identified.

Supplementary information for the outcome measures presented in each round was provided to participants in the form of a table. The information provided for each outcome measure included a general description, age range for use, an estimate of time taken to complete the measure, estimated cost, any available translations, and a link to further online resources.

### First round

Participants were presented with 20 outcome measures across four domains (pain, function, fatigue, quality of life) and were asked to rate each outcome measure as ‘unimportant’, ‘important’, or ‘essential’ for use in clinical practice when assessing paediatric patients with hypermobility and chronic lower limb musculoskeletal pain. At the end of the survey, participants were invited to comment in free text boxes on general considerations regarding outcome measure selection or suggest other outcome measures not already captured. Outcome measures that did not reach consensus as important or essential were excluded prior to round two. In addition, two members of the research team (VP and RQ) analysed the free text responses and added the outcome measures suggested by participants that met the scope of the Delphi survey. Categories of the common considerations participants suggested when selecting outcome measures were also collated.

### Second round

Participants received a summary of round one results, a document providing information on each outcome measure and a link to the round two survey. Participants were presented with outcome measures reaching consensus in the previous round as well as outcome measures suggested by participants in round one and asked to rate each outcome measure as ‘important’ or ‘not important’ for use in clinical practice when assessing paediatric patients with hypermobility and chronic lower limb musculoskeletal pain. Outcome measures that did not reach consensus as important were excluded from round three.

### Third round

Participants received a summary of round two results, a document providing information on each outcome measure, and a link with the round three survey. If more than one outcome measure met consensus in a domain, participants were asked to rank the individual outcome measures in order of importance for use in clinical practice when assessing paediatric patients with hypermobility and chronic lower limb musculoskeletal pain.

### Data analysis

In the first two rounds, frequencies and percentages of agreement were calculated for individual outcome measures. In round three, those domains with more than one outcome measure reaching consensus, were ranked in order of importance, with ‘one’ the most important outcome measure, through to ‘three’ the least important. Number values were assigned to the participants rankings (rank one = 3 points, rank two = 2 points, and rank three = 1 point). Two members of the research team calculated the weighted total points score to determine final overall ranking (Table 2, Supplementary 3). The outcome measure with the greatest weighted point score was considered the most important in each domain.

## Results

### Participant characteristics

Characteristics of the participants in all three rounds are summarised in Table 1. In round one, 52 health professionals from 13 countries participated, with over half of the participants comprising physiotherapists (38%) and doctors (31%). Of the initial health professionals who completed the Delphi, 22 participants (42%) had 20 years or more experience working with children with hypermobility-associated conditions, and the most common workplace setting was a hospital (41%). Forty-six participants completed round two, and 44 completed round three, representing 88% and 84% retention rates, respectively.

**Table 1:** Characteristics of the participants

	Round 1 (*n* = 52)	Round 2 (*n*= 46)	Round 3 (*n* = 44)
Participant profession *n* (%)			
Doctor	16 (31)	12 (26)	11 (25)
*Clinical geneticist*	1 (6)	1 (8)	1 (9)
*Orthopaedic surgeon*	3 (19)	2 (17)	1 (9)
*Paediatric anaesthesia/pain medicine*	1 (6)	1 (8)	1 (9)
*Paediatric rehabilitation*	3 (19)	2 (17)	2 (18)
*Paediatric rheumatologist*	8 (50)	6 (50)	6 (55)
Nurse	2 (4)	1 (2)	1 (2)
Occupational therapist	1 (2)	1 (2)	1 (2)
Physiotherapist	20 (38)	19 (41)	18 (41)
Podiatrist	12 (23)	12 (26)	12 (27)
Psychologist	1 (2)	1 (2)	1 (2)
**Sex *n* (%)**			
Female	38 (73)	33 (72)	32 (73)
Male	14 (27)	13 (28)	12 (27)
**Country *n* (%)**			
Algeria	1 (2)	1 (2)	1 (2)
Australia	29 (56)	27 (59)	25 (57)
Brazil	1 (2)	1 (2)	1 (2)
Canada	1 (2)	0	0
Ireland	1 (2)	1 (2)	1 (2)
Italy	1 (2)	1 (2)	1 (2)
Netherlands	4 (8)	2 (4)	2 (9)
Nigeria	1 (2)	1 (2)	1 (2)
United Kingdom	8 (15)	7 (15)	7 (16)
United States of America	2 (4)	2 (4)	2 (9)
Singapore	1 (2)	1 (2)	1 (2)
Sweden	1 (2)	1 (2)	1 (2)
Thailand	1 (2)	1 (2)	1 (2)
**Years of clinical experience *n* (%)**			
Less than 5	1 (2)	1 (2)	1 (2)
5–9	6 (12)	6 (14)	6 (14)
10–14	12 (23)	11 (25)	10 (23)
15–19	11 (21)	9 (20)	9 (20)
20 +	22 (42)	19 (43)	18 (41)
**Workplace healthcare setting *n* (%)**			
Combination of settings	11 (21)	10 (22)	10 (23)
Community health	3 (6)	3 (7)	3 (7)
Hospital			
*Paediatric specific*	18 (35)	14 (30)	13 (30)
*Non-paediatric specific*	3 (6)	3 (7)	3 (7)
Private practice	17 (33)	16 (35)	15 (34)

### Round 1

**Fig. 1:**
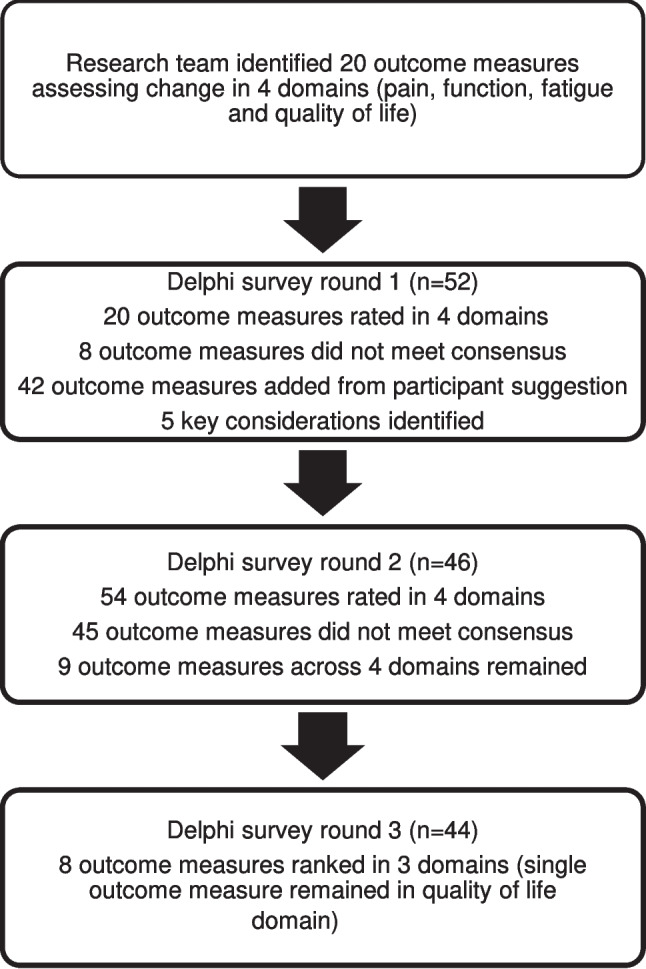
A flowchart detailing the process for reaching consensus on a core set of outcome measures

In round one, 52 of the 85 experts invited completed the survey, a response rate of 61.2%. Of the 38 who did not complete the survey, 24 did not reply to the invitation, seven declined to participate, and two returned incomplete surveys. Of the 20 outcome measures included in the survey, eight did not reach consensus as important or essential in assessing children with hypermobility and lower limb pain and were excluded (supplementary 3). After analysis of open-ended participant responses by two members of the research team, 42 further outcome measures that met the scope of the survey were added (Fig. 1). Five consideration categories for assessing children with hypermobility and lower limb pain were identified: age of the child, familiarity of the professional with the outcome measure, cost of the outcome measure, time taken to administer the outcome measure, and availability of the outcome measure in a range of languages.

### Round 2

In round two, 46 of the original 52 participants (88.5%) completed the second-round survey. Of the 54 outcome measures presented to participants, 45 were excluded as they did not reach consensus. Nine outcome measures remained across four domains: three for assessing pain, two for function, three for fatigue, and one for quality of life (Fig. 1).

### Round 3

In round three, 44 of the 46 participants (95.6%) who completed the second round responded to the round three survey. Participants ranked the outcome measures in order of importance in three of the domains (Table 2). Ranking was not required in the quality-of-life domain as the child reported Pediatric Quality of Life Inventory (PedsQL) Generic Core Scales was the only outcome measure to reach consensus.

**Table 2:** Nine outcome measures in four domains that reached consensus ranked in order of importance

Domain	Rank	Outcome measure
*Pain*	1	PedsQL-pediatric pain questionnaire (child reported)
	2	Visual analogue scale-pain (child reported)
	3	PedsQL-pediatric pain questionnaire (parent reported)
*Function*	1	Number of school days missed in last fortnight
	2	Six-minute walk test
*Fatigue*	1	PedsQL-multidimensional fatigue scale (child reported)
	2	Visual analogue scale-fatigue (child reported)
	3	PedsQL-multidimensional fatigue scale (parent reported)
*Quality of life*	1	PedsQL-generic core scales (child reported)

*PedsQL* pediatric quality of life inventory

## Discussion

This study aimed to reach consensus on an internationally relevant core set of outcome measures that can be used in clinical practice for children with hypermobility-associated conditions and chronic lower limb musculoskeletal pain. The final set comprised nine outcome measures across four domains, including pain, function, fatigue, and quality of life. We believe this is the first study to reach consensus on a set of core outcome measures to utilise in assessing change for children with hypermobility-associated conditions and chronic lower limb musculoskeletal pain.

Historically, the absence of consensus on, and considerable variability in the selection of outcome measures has posed challenges in clinical practice and research settings. Recent systematic reviews have highlighted heterogeneity in outcome measures used in research settings to be a critical limitation [3, 14–16]. Furthermore, a recent scoping review concluded there were no agreed sets of outcome measures for assessing change in children with symptomatic hypermobility [14]. Extensive variability in the selection of outcome measures amongst health professionals was demonstrated in the first round of this study when a further 42 outcome measures were suggested by participants, in addition to the 20 already identified from the literature. Despite this variability, the factors participants considered within outcome measure selection relating to patients, practitioners, and the outcome measure itself were consistent with current literature [25, 26].

Outcome measures that met consensus in this study possess characteristics recognised to increase implementation efficiency in clinical and research settings [27, 28]. All outcome measures prioritised within this current study can be administered without formal training, in under ten minutes, and require no equipment or minimal set-up. Most outcome measures (8 out of 9) are question-based and are freely available for most unfunded research and clinical purposes. Furthermore, outcome measures have minimal language barriers, with the PedsQL scales and 6MWT already utilised in multiple languages [29–32]. Minimal translation difficulties exist for the visual analogue scales and school absenteeism outcome measure.

Notably, no paediatric hypermobility condition-specific outcome measure was identified through the literature search or suggested by participants. While outcome measures that met consensus in this study do not have established reliability and validity for use in this patient population, all measures have either been extensively used and/or validated in paediatric populations with concomitant conditions [33–39]. However, since the completion of this Delphi, the Spider questionnaire, a disease-specific tool able to guide multidisciplinary management by measuring the presence and impact of multisystemic symptoms and comorbidities associated with joint hypermobility, has been validated [40]. Convergent validity of the Spider has been established for both adolescents and adults with hypermobility-associated conditions [40, 41]. Furthermore, while only validated in adults, the Bristol Impact of Hypermobility questionnaire measures the impact of hypermobility-associated conditions on an individual’s quality of life. Consideration of development and validation of a paediatric version of this tool is warranted [42].

This study supports clinicians and researchers selecting outcome measures that prioritise the child’s perspective. Child-reported outcome measures were ranked above parent-reported in the pain and fatigue domains, where both had reached consensus. Furthermore, a child-reported outcome measure was the only measure to meet consensus in the quality-of-life domain. For most outcome measures, scant evidence exists to inform clinician decisions on utilising a child or parent report [43–45]. Clinicians therefore may rely on professional judgement and any recommendation from the outcome measure developer; for example, the PedsQL scales are considered appropriate for children 5 years and older; however, parent proxy is recommended until 8 years of age [43]. Further complicating decision making are the unique ethical and practical considerations for clinicians in paediatric settings, such as consent, privacy, and the cognitive ability of individual children [27, 46]. However, emerging evidence recommends inclusion of children in self-reporting their own HRQoL and pain wherever possible and limiting the reliance on proxy reporting [46, 47]. This is particularly important for children with hypermobility-associated conditions, as no agreement on HRQoL has been demonstrated in current literature, and most concerningly, parents have typically underreported their child’s pain [13, 48].

There are limitations to this study with low representation of participants from certain health professions, such as nursing, occupational therapy, and psychology. However, health professions participating in this panel were from 13 countries and represented members commonly constituting multidisciplinary teams involved in the gold standard management of children with hypermobility-associated conditions [13, 49]. Secondly, only English-speaking participants were included, with almost 70% of participants from high-income countries, as classified by the World Bank, confirming outcome measure use in developing countries may require further consensus in future research [28, 50]. Finally, while it is recommended that typical Delphi panels consist of up to 100 members, this Delphi panel of 54 participants is significant given the specificity of the patient population and the demonstrated high rate of retention throughout all rounds [24].

An international consensus of health professionals has determined that the most important outcome measures to use in the assessment of children with hypermobility-associated conditions and chronic musculoskeletal lower limb pain in the respective domains of pain, function, fatigue, and quality of life are the PedsQL Pediatric Pain Questionnaire (child reported), School absenteeism: Number of school days missed within the last fortnight, the PedsQL Multidimensional Fatigue Scale (child reported), and the PedsQL Generic Core Scales (child reported). Health professionals managing chronic lower limb pain in this patient population are encouraged to utilise this core set of outcome measures in clinical practice, and implementation in research settings could be beneficial. This consensus-based approach for determining a set of outcome measures to assess change over time represents a step toward the development of international guidelines for the management of children (5 years and older) with hypermobility and chronic lower limb pain.

## Data Availability

The data that support the findings of this study are available upon request from the corresponding author, RQ. The data are not publicly available because they contain information that could compromise the privacy of research participants.
